# Endogenous Protection Derived from Activin A/Smads Transduction Loop Stimulated via Ischemic Injury in PC12 Cells

**DOI:** 10.3390/molecules181012977

**Published:** 2013-10-17

**Authors:** Jing Mang, Chun-Li Mei, Jiao-Qi Wang, Zong-Shu Li, Ting-Ting Chu, Jin-Ting He, Zhong-Xin Xu

**Affiliations:** 1 Department of Neurology, China-Japan Union Hospital, Jilin University, Changchun 130012, China; E-Mails: mangjing@jlu.edu.cn (J.M.); wangjiaoqi111@sina.com (J.-Q.W.); Hyuliu198@163.com (H.-Y.L.); studented3@sina.com (G.-H.X.); jiangyan1984@163.com (J.Y.); bxq1966@163.com (X.-Q.B.); 2 College of Nursing, Beihua University, Jilin City 132013, China; E-Mail: meixiaoqing2007@126.com; 3 People’s Hospital of Jilin Province, Changchun 130021, China; E-Mail: Lizongshu111@sina.com; 4 The 4th Hospital of Harbin Medical University, Harbin 150006, China; ctting1986@gmail.com

**Keywords:** ischemic injury, OGD, activin A, ActA/Smads pathway

## Abstract

Activin A (ActA), a member of transforming growth factor-beta (TGF-b) super- family, affects many cellular processes, including ischemic stroke. Though the neuroprotective effects of exogenous ActA on oxygen-glucose deprivation (OGD) injury have already been reported by us, the endogenous role of ActA remains poorly understood. To further define the role and mechanism of endogenous ActA and its signaling in response to acute ischemic damage, we used an OGD model in PC12 cells to simulate ischemic injury on neurons *in vitro*. Cells were pre-treated by monoclonal antibody against activin receptor type IIA (ActRII-Ab). We found that ActRII-Ab augments ischemic injury in PC12 cells. Further, the extracellular secretion of ActA as well as phosphorylation of smad3 in PC12 cells was also up-regulated by OGD, but suppressed by ActRII-Ab. Taken together, our results show that ActRII-Ab may augment ischemic injury via blocking of transmembrane signal transduction of ActA, which confirmed the existence of endogenous neuroprotective effects derived from the ActA/Smads pathway. ActRIIA plays an important role in transferring neuronal protective signals inside. It is highly possible that ActA transmembrance signaling is a part of the positive feed-back loop for extracellular ActA secretion.

## 1. Introduction

Ischemic stroke is one of the leading causes of death, serious and long-term disability [1]. It results in oxygen and glucose deprivation of neurons and leads to necrotic loss [2]. This kind of injury can trigger endogenous activation of some neuroprotective factors and related signal transduction. Recent attention has focused on the function and mechanism of cellular signal transductions involved in the ischemic brain injury [3,4].

ActA is a member of the transforming growth factor-beta (TGF-β) superfamily, and its signaling is an important regulator of many cellular process. ActA can bind with ActRIIA and initiate the intracellular transmission of signals [5,6]. After ligand binding, ActRIIA phosphorylates and thereby activates and phosphorylates ActRIA, as well as intracellular R-Smads (Smad2/3). Then the latter forms a heterodimer, which will regulate target genes by translocating into the nucleus and recruiting transcriptional co-activators or co-repressors [7,8,9].

It has been reported that exogenous ActA can protect neurons against degeneration damage as well as ischemic injury [10,11,12]. However, whether there is an endogenous ActA/Smads pathway involved in brain ischemic insult was rarely documented. Based on an OGD model of PC12 cells, we report that ActR-Ab can block the ActA transmembrane signal as well as augment ischemia injury in PC12 cells. Through investigating the expression changes in basic components of the ActA/Smads pathway, our results elucidated the effects of endogenous ActA on neuron-like cells subjected to ischemic damage *in vitro*, which also brought novel insights into our understanding of the transduction loop of ActA/Smads signaling in model ischemic brain injury *in vitro*.

## 2. Results and Discussion

### 2.1. MTT Assay of PC12 Cells Subjected to OGD Injury

**Figure 1:**
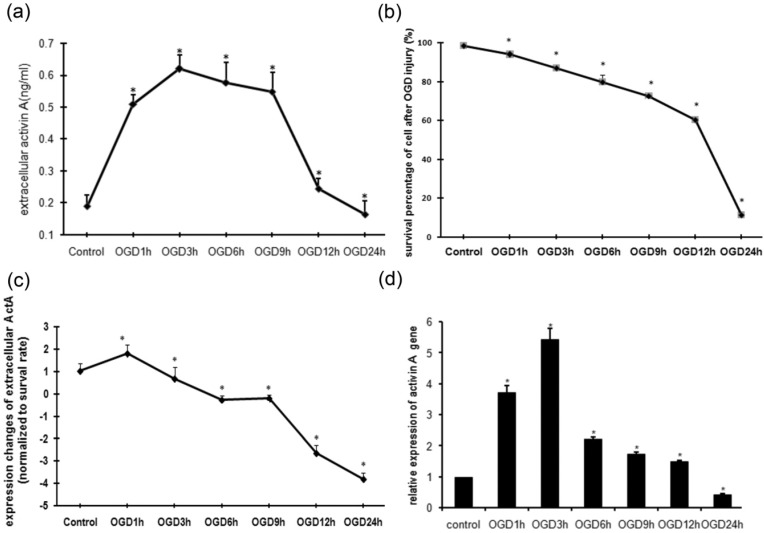
OGD injury stimulated up-regulation of ActA expression in PC12 cells. (**a**) MTT assay of cells exposed to OGD injury. The survival rates of PC12 cells after different time of OGD injury were measured by an MTT assay compared to the control group. (**b**) Concentration of extracellular ActA with different OGD exposure times. PC12 cells in the control group produce only a minute amount of ActA (0.189 ± 0.035 ng/mL). As the OGD time is extended the concentration of ActA is increased and reaches its peak at 3 h (0.619 ± 0.043 ng/mL), then declines gradually. (**c**) Expression changes of extracellular ActA due to OGD injury. ActA expression in each time point was measured, subtracted from the previous monitoring point and compared to the survival rate. The amount of cellular autocrined ActA in the OGD 1 h group was the highest. (**d**) Expression of ActA mRNA changed correspondingly with different OGD exposure time, in the same manner as the extracellular ActA. (*: compared to control group, *p* < 0.05).

The ischemic damage to brain tissue during cerebral infarction was simulated by OGD injury caused in PC12 cells through oxygen and glucose deprivation. The survival rates of cells after different times of OGD exposure were measured by an MTT assay compared to the control. As shown in Figure 1a, the cell survival percentages after 1 h or 3 h of OGD damage were 93.87 ± 1.34% and 86.82 ± 1.25%, respectively. As the OGD exposure was extended the rate of cell survival declined, especially after 12 h of OGD treatment.

### 2.2. OGD Injury Up-Regulated the Production of ActA by PC12 Cells

A large body of evidence has shown that various factors take part in the neuroprotective effect in the process of ischemic damage, but their mechanisms and signal pathways are not the same. For example, Neuregulin-1 beta can activate the JAK/STAT signal transduction pathway, promote astrocyte gumnosis and regulate the anti-apoptosis mechanism in neurocytes [13]. Phosphorylation of p38 mitogen-activated protein kinase (p38 MAPK) mediates hypoxic preconditioning-induced neuroprotection against cerebral ischemic injury via mitochondria translocation of Bcl-xL [14]. Some even play a double role in cerebral ischemia, like IL-6, which acts as an inflammatory mediator during the acute phase and as a neurotrophic mediator between the subacute and prolonged phases [15].

As an autocrine factor in many biological systems, the production and secretion of ActA in neuron-like cells are not clear [16,17]. The PC12 cell line derived from rat adrenal pheochromocytomas has been intensively used for studies in neurobiology [18,19]. In this study a low level of ActA expression was detected in the culture medium of normal PC12 cells (Figure 1b). As the OGD time was extended, the extracellular protein as well as intracellular mRNA expression of ActA increased significantly, reaching its peak at 3 h (Figure 1b,c), then declined gradually. Our findings suggest that OGD injury could dynamically activate both the extracellular secretion and intracellular expression of ActA. The up-regulation of extracellular ActA may be an endogenous response to acute ischemic (OGD) injury. The extracellular level of ActA in the medium of PC12 cells cultured in Petri dish was a dynamic process of autocrine signaling and consumption by binding to the receptor, and it was affected by the cell survival rate after OGD injury, so the extracellular expression changes of ActA due to OGD exposure were normalized by the survival rate at each time point. As shown in Figure 1d, the remaining ActA was dramatically increased after 1 h of OGD injury, and declined gradually with the extension of OGD exposure. This phenomenon suggested that the expression of Act was activated at the early time of OGD injury. There is an accumulation of ActA with the decrease of available receptor. At the 6 h and 9 h OGD exposure timepoints, the secretion and consumption of ActA were almost at the same level, and then the ActA product was significantly decreased due to the death of cells after the long duration of the OGD injury. During this process the expression of ActA was activated, redundant and accumulated at a short time of OGD injury (3 h). Therefore, there may be a positive feedback loop linking ActA intracellular signaling to the outer secretion of ActA, and it appears in the early (acute) stage of OGD (ischemic). The function derived from activation of ActA and its downstream signaling may therefore be potential therapeutic targets for the treatment of ischemic brain diseases.

### 2.3. ActRⅡ-Ab Suppressed both Intracellular Signaling and Extracellular Secretion of ActA in PC12 Cells Subjected to OGD

**Figure 2:**
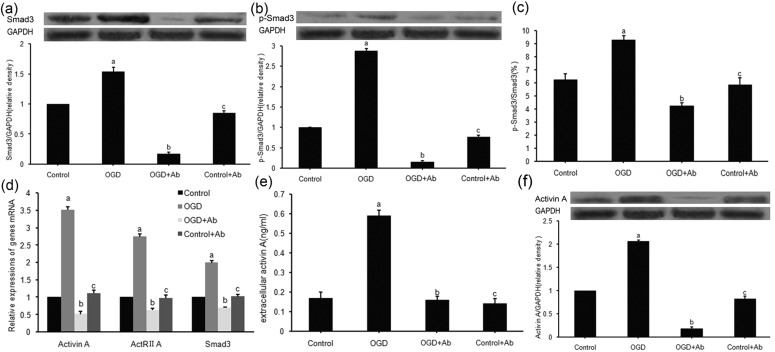
Effects of ActRII-Ab on ActA transmembrance signaling and extracellular secretion. (**a**, **b**) Smad3 (**a**) and p-Smad3 (**b**) protein was up-regulated (53.9 ± 7.1% and 187.4 ± 6.8% respectively) by 3 h OGD, but was inhibited (88.9 ± 3.4% and 94.9 ± 4.5% respectively) by ActRII-Ab. (**c**) 3 h OGD stimulated a 48.9 ± 7.2% increase in phosphorylation level of Smad3, but was inhibited 54.4 ± 4.2% by ActRII-Ab. (**d**) ActA, ActRIIA and Smad3 genes were up-regulated due to 3 h OGD (251.6 ± 9.7%,174.2 ± 6.2% and 99.6 ± 7.0%, respectively), but inhibited (85.4 ± 8.2%,77.2 ± 6.3% and 64.9 ± 2.2% respectively) by ActRII-Ab. (**e**,**f**) The ActRII-Ab decreased the extracellular secretion of ActA protein by 73.2 ± 2.1% (**e**) and intracellular expression by 91.3 ± 3.7% (**f**). (a: compared to control group, *p* < 0.05; b: compared to OGD group, *p* < 0.05; c: compared to control group, *p* > 0.05).

The ActA-triggered Smads signaling pathway plays an important role in many cellular processes [11,12], but the endogenous mechanisms of the ActA/Smads pathway involved in ischemic brain injury remain unclarified. In this study, a previously undefined ActA/Smads pathway inhibitor (ActRII-Ab) was introduced to the OGD model of PC12 cells. To determine the effect of ActRII-Ab on ActA signaling and make further understanding of changes of ActA signal transduction during ischemic damage, the expressions of ActA, ActRIIA and Smad3 were detected. The results demonstrated that OGD injury significantly up-regulated these three genes, compared to control group (Figure 2d). Then the activator (ActA) and downstream transcription factors (Smad3 and p-Smad3) of the ActA/Smads pathway were detected. Both the extracellular (Figure 2e) and intracellular (Figure 2f) expression of ActA protein increased due to OGD exposure, but the ActR-Ab significantly suppressed the up-regulation of ActA induced by OGD (Figure 2e,f). In a similar manner, the downstream transcription factors (Smad3 and p-Smad3), and the phosphorylation ratio of Smad3 were also activated by OGD, but suppressed by ActRII-Ab (Figure 2a–c).

These findings reaffirmed that a short-time ischemic injury (3 h OGD) may stimulate the activation of ActA/Smads pathway [12]. Moreover, as we confirmed above, PC12 cells produce ActA in an autocrine manner, so our results suggest that increased extracellular ActA may play a dual role in the ActA/Smads pathway: it is both the initial activator and the finally product of the pathway. All of the above implies a positive feed-back loop for intercellular ActA signaling and extracellular ActA secretion of PC12 cells subjected to ischemic (OGD) injury. We reasoned that, ischemic neurons were the producer and target of ActA, the ActA autocrined by neurons act with a “target-derived” pattern in signal transduction. Similar mechanisms exist in many TGF-β signaling [20,21], but never defined in neuron like cells through the process of ischemia *in vitro*.

Methodologically, we found up-regulation of the main factors (ActA, ActRIIA and p-Smad3/Smad3) induced by OGD was significantly suppressed by ActRII-Ab, which suggests the blockade of ActA signaling. These findings indicate a novel and easy strategy for the investigation of the ActA signaling pathway.

### 2.4. ActRII-Ab Augments Injury in PC12 Cells Induced by Ischemia (OGD) via Blocking of Activin A Signaling

**Figure 3:**
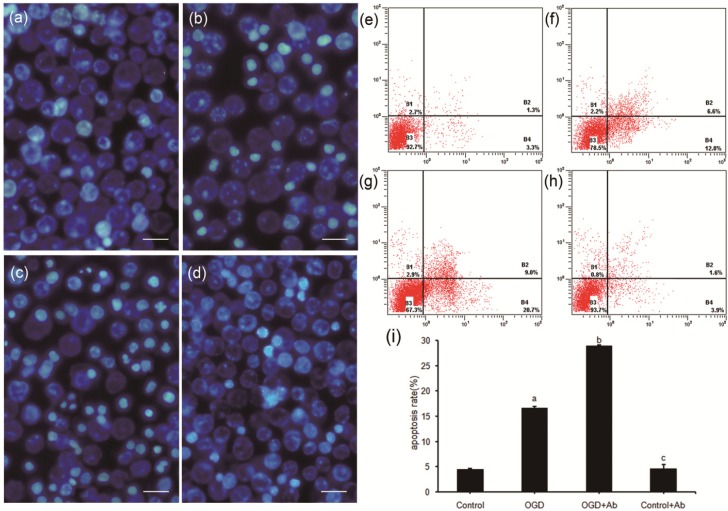
ActRII-Ab augmented injury in PC12 cells subject to 3 h OGD. (**a**–**d**) Morphological changes of apoptotic cells detected by Hoechst 33342 staining (bar = 50 μm). (**a**) Control group, no severe morphological changes of apoptosis. (**b**) OGD group, few apoptotic cells with stronger blue fluorescence or chromatin condensation. (**c**) OGD+Ab group, more apoptotic cells were found. (**d**) Control+Ab group, no significant difference compared to Control group. (**e**–**i**) Apoptotic rate determined by flow cytometry analysis with the Annexin V/PI double staining assay. Early apoptotic cells were shown in the lower right quadrant, late apoptotic cells in the upper right quadrant. Figures represent one sample of three independent experiments. (**e**) Control group. (**f**) OGD group. (**g**) OGD+Ab group. (**h**) Control+Ab group. (**i**) Apoptotic rate in OGD group was higher than Control group (16.62 ± 0.78% *vs.* 4.47 ± 0.13%, a: *p* < 0.05), which was even increased in OGD+Ab group (28.96 ± 0.92% *vs.* 16.62 ± 0.78%, b: *p* < 0.05). There was no significant difference between Control and Control+Ab group (4.47 ± 0.13% *vs.* 4.58 ± 0.85%, c: *p* > 0.05).

The neuroprotective effects of recombinant ActA have been approved in brain ischemia [12,22], but whether there exist similar effects of endogenous ActA induced by ischemia injury *in vitro* are not clear. In the present study, with the presence of ActRIAb, the damage due to OGD injury was found to be augmented in PC12 cells (Figure 3), accompanied with the blockade of the ActA/Smads signaling. Our results provide strong evidence for the protective role of endogenous ActA for ischemic neurons.

In the transduction of the ActA/Smads pathway, ActA need to bind with the transmembrane receptor to activate the signaling [7,8]. After ActRII was blocked by ActRII-Ab, the activation of the pathway was suppressed, the expressions of ActA and Smad3 were down-regulated, suggesting that endogenous activation of ActA/Smads pathway may be initiated by the binding of extracellular ActA and its receptor, and ActRIIA is crucial in the cascade signal of ActA/Smads pathway required for neuroprotection. However, as the mediator of the ActA/Smads pathway, the dynamic changes of the pathway may be due to the binding capacity of ActRIIA which was regulated by the different course time of ischemia.

## 3. Experimental

### 3.1. Cell Culture and Oxygen-Glucose Deprivation

Rat adrenal pheochromocytoma (PC12) cell line was obtained from Gold Amethyst Pharm & Bio-Tech Co. Ltd (Beijing, China). Cells were grown in complete Dulbecco’s modified Eagle’s medium (DMEM, Gibco, Carlsbad, CA, USA) supplemented with 15% fetal bovine serum (FBS, Gibco), in a humidified atmosphere of 5% CO2 and 95% air at 37 °C, subcultured every 4–5 days routinely. Glucose-free DMEM (Gibco) supplemented with deoxygenated 1.0 mmol/L sodium dithionite (Na_2_S_2_O_4_) was used for the induction of OGD as previously described [23]. PC12 cells seeded at a density of 1.0 × 10^8^/L were washed three times and then cultured in the OGD medium at hypoxic conditions (37 °C, 5% CO_2_ and 95% N_2_).

### 3.2. Cell Survival Rate Assay

Cells were plated in 96-well plate and treated with OGD for 0, 1, 3, 6, 9, 12 and 24 h. Every group had five parallel wells. The group of OGD 0 h was used as control, and the blank well as blank control. At the end of the treatment, MTT (20 μL, 5 mg/mL in PBS, Sigma, St. Louis, MO, USA) was added to each well. After 4h of incubation, the media were removed and DMSO (200 μL) was added into each well. Then the 96-well plate was carefully shaken for 5 min to dissolve the blue formazan product. The optical absorbance (*A*) of each well was read using a Universal Microplate reader (Bio-TEK Instrument. Inc., Winooski, VT, USA) spectrophotometer at 490 nm. The percentage of survival cells in each group was calculated as follows: (*A* of OGD X h experimental group − *A* of blank control group)/(*A* of OGD 0 h group − *A* of blank control group) × 100%.

### 3.3. Extracellular Level of ActA Assay by ELISA

Extracellular concentration of ActA in culture medium was measured using ActA ELISA kits (E90001Ra, Uscn, zoersel, Belgium) according to the manufacturer’s instructions. The exposure time of cells to OGD treatment was 0, 1, 3, 6, 9, 12, 24 h. The OGD 0 h group was acted as control. Optical absorbance (*A*) of each well was read with microplate reader (Bio-TEK Instrument Inc.) spectrophotometer at 450nm. To compare results from different plates, absorbance (*A*) in each sample was adjusted relative to the positive and negative Standard Diluent supplied in each kit. The index value of each tested serum was defined by the following formula: index = (*A* of tested cell culture − *A* of negative control)/(*A* of positive control − *A* of negative control) × 100. Then, ActA expression in each time point were subtracted to the former watch point and compared to the survival rate by the MTT assay, after normalization to the extracellular ActA expression in the Control group.

### 3.4. Pre-Treatment of ActRⅡ-Ab

PC12 cells were plated in 6-well plates at a density of 1.0 × 10^8^/L. Cells were incubated with 25μg/mL ActRII monoclonal antibody (A0856-05E, US Biological, Samel, MA, USA) for 12 h prior to OGD. And there were four groups: normal PC12 cells without any treatment (Control group), ActRII-Ab treated PC12 cells (Control+Ab group), PC12 cells exposed to OGD (OGD group) and ActRII-Ab pre-treated PC12 cells exposed to OGD (OGD+Ab group). The exposure time of OGD treatment was 3 h.

### 3.5. Hoechst33342 Fluorescence Staining

Cells were plated in 8-well chamber slides (Thermo Scientific, Waltham, Massachusetts, USA) at a density of 2.5 × 10^7^/L, and then they were randomly separated into four groups and treated as above. To analyze morphological changes associated with apoptosis, Hoechst 33342 (5 μg/mL, Invitrogen, Carlsbad, CA, USA) was then added to each chamber and incubated for 60 min at 37 °C before fluorescence microscopic analysis (Olympus BX61, Tokyo, Japan).

### 3.6. Flow-Cytometry Analysis

Cells in those four groups were harvested and stained by Annexin V-FITC/PI double staining kit (Invitrogen) according to the manufacturer’s instructions. Then they were detected by flow cytometry (FCM, Beckman Coulter FC 500, Laguna Woods, CA, USA).

### 3.7. Real-Time PCR

Real-time PCR based on SYBR-Green I was performed using 7500 Fast system (ABI, Foster City, CA, USA). The sequences of primers for ActβA, ActRIIA, Smad3 and GAPDH genes were as follows: ActβA, U:5′-TAG TTT ACC TGG GAT GAA GC-3′ and D:5′-TAG CAC CCT CTA ACA CCT CT-3′, ActRIIA, U:5′-ATG TCA TCT ACT GCC GCT TGT GG-3′ and D:5′-ATG CTG TGG TTC ATC TGG TGG TC-3′, Smad3, U:5′-ATG TCA TCT ACT GCC GCT TGT GG-3′ and D:5′-ATG CTG TGG TTC ATC TGG TGG TC-3′, GAPDH, U:5′-GCA GTG GCA AAG TGG AGA TT-3′ and D:5′-TGT CTT CTG GGT GGC AGT GAT-3′. Total RNA was extracted using TrizolPlus Columns kit (Invitrogen). Synthesis of cDNA was carried out with PrimeScript 1st Strand cDNA Synthesis Kit (TaKaRa, Tokyo, Japan). A 20 μL reaction system of SYBR PrimeScript RT-PCR Kit II (TaKaRa) was established according to the manufacturer’s instructions.

### 3.8. Western Blot Analysis

Western blot detection was performed as described previously [13]. The primary antibodies were as follows: anti-smad3 (Santa, rabbit, SC-101154), anti- Phospho-Smad3 (Santa, rabbit, SC-11769), anti-ActA (Santa, rabbit, sc-50288), anti-GAPDH (Santa, rabbit, sc-25778). The immunoblot was revealed with an ECL western blotting detection kit (Amersham Pharmacia Biotech, Buckinghamshire, England). Densitometry analysis was performed using ImageJ software (National Institutes of Health, Bethesda, MD, USA).

### 3.9. Statistical Analysis

All experiments were carried out three times on different samples. Values were presented as the means ± S.E. of (n) determinations. Differences among groups (more than 2) were determined by using analysis of variance (ANOVA), and Student’s *t* test was used for single comparison between two groups by Sigma Stat statistical software package (SPSS 13.0, International Business Machines Corporation, Chicago, IL, USA). A two-tailed probability of less than 5% (*i.e.*, *p* < 0.05) was considered statistically significant.

## 4. Conclusions

In conclusion, ischemic injury induced by OGD can activate the ActA/Smads signaling pathway. A positive feed-back loop for the activation of ActA signaling may exist through the process of ischemic (OGD) injury *in vitro.* Blockade of ActA Signal transduction augments OGD damage, which suggests the existence of endogenous neuroprotective effects derived from the ActA/Smads pathway. However, in particular, the networks and cross talk of various signaling pathways regulated by ActA remains to be examined in brain ischemia injury.
